# Impact of Polychlorinated Biphenyls Contamination on Estrogenic Activity in Human Male Serum

**DOI:** 10.1289/ehp.7745

**Published:** 2005-05-26

**Authors:** Martina Plíšková, Jan Vondráček, Rocio Fernandez Canton, Jiřií Nera, Anton Kočan, Ján Petrík, Tomáš Trnovec, Thomas Sanderson, Martin van den Berg, Miroslav Machala

**Affiliations:** 1 Veterinary Research Institute, Brno, Czech Republic; 2 Institute of Biophysics, Czech Academy of Sciences, Brno, Czech Republic; 3 Institute of Risk Assessment Sciences, University of Utrecht, Utrecht, the Netherlands; 4 Slovak Medical University, Bratislava, Slovakia

**Keywords:** CYP1A1, CYP1B1, dioxin-like activity, estradiol, estrogenicity, human serum, polychlorinated biphenyls

## Abstract

Polychlorinated biphenyls (PCBs) are thought to cause numerous adverse health effects, but their impact on estrogen signaling is still not fully understood. In the present study, we used the ER-CALUX bioassay to determine estrogenic/antiestrogenic activities of the prevalent PCB congeners and PCB mixtures isolated from human male serum. The samples were collected from residents of an area with an extensive environmental contamination from a former PCB production site as well as from a neighboring background region in eastern Slovakia. We found that the lower-chlorinated PCBs were estrogenic, whereas the prevalent higher-chlorinated PCB congeners 138, 153, 170, 180, 187, 194, 199, and 203, as well as major PCB metabolites, behaved as anti-estrogens. Coplanar PCBs had no direct effect on estrogen receptor (ER) activation in this *in vitro* model. In human male serum samples, high levels of PCBs were associated with a decreased ER-mediated activity and an increased dioxin-like activity, as determined by the DR-CALUX assay. 17β-Estradiol (E_2_) was responsible for a major part of estrogenic activity identified in total serum extracts. Significant negative correlations were found between dioxin-like activity, as well as mRNA levels of cytochromes P450 1A1 and 1B1 in lymphocytes, and total estrogenic activity. For sample fractions containing only persistent organic pollutants (POPs), the increased frequency of anti-estrogenic samples was associated with a higher sum of PCBs. This suggests that the prevalent non-dioxin-like PCBs were responsible for the weak antiestrogenic activity of some POPs fractions. Our data also suggest that it might be important to pay attention to direct effects of PCBs on steroid hormone levels in heavily exposed subjects.

Polychlorinated biphenyls (PCBs) are a group of structurally diverse and persistent environmental pollutants, widely distributed as complex mixtures. Mechanisms of toxicity of individual PCB congeners depend on the planarity of a molecule (Safe 1994), as well as on molecular weight and biotransformation rate (Rose et al. 2002). Similarly to 2,3,7,8-tetrachlorodibenzo-*p*-dioxin (TCDD), the coplanar non-*ortho*-substituted PCBs activate aryl hydrocarbon receptor (AhR) and AhR-dependent signal transduction pathways (van den Berg et al. 1998). A majority of the adverse effects of these compounds is thought to be mediated through AhR activation. Therefore, the toxic potencies of dioxin-like PCBs can be expressed in terms of toxic equivalency factors (TEFs) relative to TCDD as the reference toxicant. The TEF values of individual PCBs multiplied by their respective concentrations can be used to yield TCDD toxic equivalents (TEQs) (van den Berg et al. 1998). In contrast, a distinct set of AhR-independent effects, including neurotoxicity, (anti)estrogenicity, and tumor promotion, has been found after exposure to noncoplanar *ortho*-substituted PCBs (Brouwer et al. 1999; Hansen 1998; Machala et al. 2003; Robertson and Hansen 2001); however, the modes of action of nondioxin-like PCBs are often not clear.

The biological activities of PCBs have been reported to include both estrogenic and anti-estrogenic effects in various *in vitro* and *in vivo* models (Cooke et al. 2001; Hansen 1998). TCDD and other AhR agonists, including dioxin-like PCBs, have been frequently reported to have antiestrogenic activity (Buchanan et al. 2000, 2002; Oenga et al. 2004; Safe and Wörmke 2003). Several modes of antiestrogenic action of AhR agonists might include repression of 17β-estradiol (E_2_)-dependent gene expression by interactions of activated AhR with DNA regions of E_2_ responsive gene promoters (see Oenga et al. 2004, Safe and Wörmke 2003), inhibition of E_2_-induced cell cycle proteins and uterine epithelial mitogenesis (Buchanan et al. 2002; Wang et al. 1998), or effects of PCBs on E_2_ metabolism (Pang et al. 1999; van Duursen et al. 2003). In contrast, the exact mechanisms of estrogenic or antiestrogenic activities of nondioxin-like PCBs are still not fully characterized. The reported results are often contradictory, derived from data obtained in different *in vitro* or *in vivo* models (Hansen 1998). The majority of studies found that low-molecular-weight PCBs elicit estrogenic activity both *in vitro* and *in vivo* (Arcaro et al. 1999; Nesaretnam and Darbre 1997; Rogers and Denison 2000; Rose et al. 2002). In contrast, the three most prevalent nondioxin-like PCBs, 2,2′,3,4,4′,5′-hexachlorobiphenyl (PCB 138), 2,2′,4,4′,5,5′-hexachlorobiphenyl (PCB 153), and 2,2′,3,4,4′,5,5′-heptachloro-biphenyl (PCB 180), have been reported to be antiestrogenic in MCF-7 cells (Bonenfeld-Jorgensen et al. 2001). However, estrogenic/ antiestrogenic potencies of a large set of PCB congeners have not yet been determined in a single *in vitro* bioassay. Taken together, there is only limited information on effects of prevalent nondioxin-like PCBs and complex PCB mixtures in mammalian blood and tissues.

One essential question is whether the chronic exposure to low doses of environmental persistent organic pollutants (POPs), including PCBs, has endocrine-disrupting effects on exposed human populations (Brouwer et al. 1999; Daston et al. 2003). There are only limited data on estrogenic and dioxin-like activities of complex samples of organic compounds collected from human blood. Sonnenschein et al. (1995) and Soto et al. (1997) reported the development of a serum extraction method for separation of POPs and endogenous steroids. Recently, this extraction and fractionation technique has been adapted for combined chemical and *in vitro* assay analysis in human blood, allowing for discrimination of effects of endogenous hormones and xenoestrogens (Fernandez et al. 2004). However, results of direct measurements of estrogen receptor (ER)-mediated activity of serum extracts or total POPs fractions in a comprehensive set of human subjects have not yet been published. More information is available concerning *in vitro* bioassays of dioxin-like activity in human blood contaminated with PCBs. The total TEQ values determined in human female serum and follicular fluid by the DR-CALUX (dioxin receptor–chemically activated luciferase expression) assay have been reported to correlate well with the sum of four major PCB congeners: 153, 138, 180, and 118 (Pauwels et al. 2000). The possible impact of environmental endocrine disruptors on breast cancer, male reproductive tract problems, or prostate cancer is questionable (Chen et al. 2003; Safe 2004). Nevertheless, estrogens play a significant role in, for example, testicular function (O’Donnell et al. 2001). Because the levels of endogenous estrogens in males are considerably lower than in females, possible estrogenic/ antiestrogenic impact of high levels of contamination could be more pronounced in males. Therefore, determination of *in vitro* estrogenic/antiestrogenic activities of extracts of human male blood samples collected from a PCB-contaminated area could yield more information about the impact of PCBs and/or other POPs on estrogen-dependent signaling.

Since 1959, several thousand tons of residues from the Chemko Strážske chemical plant in the Michalovce district, Slovakia, have been deposited in the nearby river and water reservoir sediments. This has resulted in widespread contamination of the environment, leading to high human exposure. Serum PCB concentrations in subjects from six different districts of Slovakia suggest that levels are three to six times higher in subjects from the Michalovce district (Kočan et al. 2001). When serum levels of 15 PCBs were compared in residents of two districts in eastern Slovakia, one with extensive environmental contamination from a former PCB production site (Michalovce) and the other matched on geography but with background PCB levels, the age-adjusted geometric means for the sum of 15 measured PCB congeners were statistically significantly higher in subjects from the Michalovce district for both sexes: 3327.6 versus 1331.4 ng/g lipid in males, 2751.8 versus 992.2 ng/g lipid in females (Pavúk et al. 2004).

As a part of a large epidemiologic study, the PCBRisk project (Trnovec et al. 2000), we investigated effects of extensive contamination with PCBs on human serum dioxin-like, estrogenic, and antiestrogenic activities of serum extracts from subjects living in the contaminated area. In this study, the ER-mediated activities of individual PCB congeners, which were identified as principal contaminants present in serum of human population in the studied area, were investigated using the T47D breast cancer cell line stably transfected with the luciferase reporter gene under control of estrogen-responsive elements, detecting the direct activation of ER (the ER-CALUX assay) (Legler et al. 1999). In the second step of the study, effects of chronic PCB exposure on antiestrogenic/estrogenic and dioxin-like activities exerted by extracts of human male sera (150 human male serum samples) were assessed and compared to concentrations of major POPs and levels of E_2_ in serum.

## Materials and Methods

### Chemicals.

**Figure 1:**
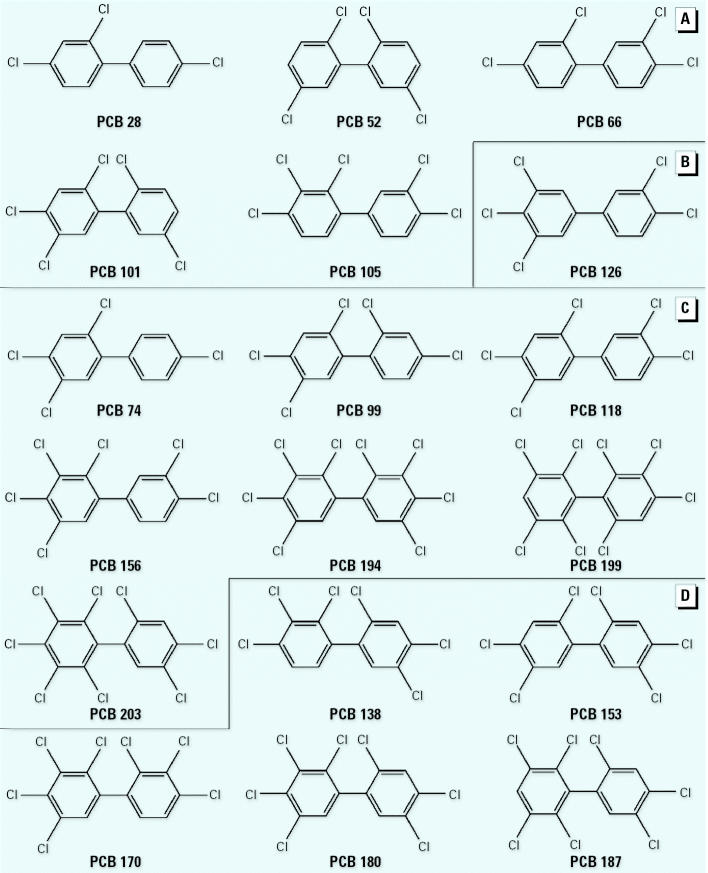
Chemical structures of selected PCB congeners examined for the antiestrogenic/estrogenic activities in human breast carcinoma T47D.Luc cells (ER-CALUX assay). (*A*) Lower molecular-weight PCBs present in low concentrations in male blood samples. (*B*) Non-*ortho*-chlorinated PCB. (*C*) PCB congeners occurring in relatively higher concentrations in male blood. (*D*) Prevalent high-molecular-weight PCB congeners.

The PCB nomenclature used here is from the International Union of Pure and Applied Chemistry (IUPAC). PCBs 74, 156, 170, 187, 199, and 203 were purchased from Ehrenstorfer (Augsburg, Germany); PCBs 28, 52, 66, 99, 101, 105, 118, 126, 138, 153, 180, and 194 were supplied by Promochem (Wesel, Germany). Purity of all compounds was > 99%. The chemical structure and nomenclature of the PCB congeners we studied is presented in Figure 1. TCDD was supplied by Cambridge Isotope Laboratories, (Andover, MA, USA); E_2_, cell culture media, and solvents were obtained from Sigma-Aldrich (Prague, Czech Republic). Stock solutions were prepared with dimethyl sulfoxide (DMSO) and stored in the dark. The final concentrations of solvent in the medium did not exceed 0.2% (vol/vol).

### Blood sampling, extraction, and clean up.

We collected 150 individual male blood samples from residents of two areas of eastern Slovakia, which are differently contaminated with PCBs: the Michalovce district, where commercial PCB mixtures were produced for a number of years (Kočan et al. 2001), and the Stropkov district, which represented the background area. The samples of human male serum (5 mL) were treated with 2 mL methanol and extracted three times with *n*-hexane:diethyl ether (1:1); the extracts were evaporated and dissolved in 1 mL dichloro-methane (Horander et al. 2004). For determination of overall ER-mediated activity, we replaced the solvent with DMSO in one-half of the crude extract; the second half of the sample was placed on a sulfuric acid-activated silica column and eluted with *n*-hexane:diethyl ether mixture, evaporated, and redissolved in DMSO (Murk et al. 1997). Using these experimental settings, only persistent compounds were eluted, including PCBs and polychlorinated dibenzo-*p*-dioxins (PCDDs) and dibenzofurans (PCDFs).

### Chemical analysis of POPs.

We determined concentrations of prevalent (non-coplanar) PCB congeners, hexachlorobenzene, and *p,p*′-DDE by gas chromatography/mass spectrometry (GC/MS) (Kočan et al. 2001, 2004). We calculated TEQs from high performance GC/MS data on blood concentrations of PCDD/PCDFs and non-*ortho*- and mono-*ortho*-chlorinated PCBs. The sum of PCBs (∑PCBs) used in the correlation and multivariate statistical analysis was based on the data on concentrations of 17 indicator coplanar and mono-*ortho*-chlorinated PCB congeners, including PCBs 28, 52, 66, 74, 77, 99, 101, 105, 118, 126, 138, 153, 156, 169, 170, 180, and 189.

### Determination of effects associated with AhR activation.

We determined the levels of cytochrome P450 (CYP) 1A1 and CYP1B1 mRNA in human peripheral lymphocytes by RNA extraction and a quantitative reverse-transcriptase-polymerase chain reaction (RT-PCR) method using TaqMan technology (Canton et al. 2003; van Duursen et al. 2005). The *in vitro* potencies of POPs present in serum to activate AhR were measured in sulfuric acid/silica-treated extracts by a luciferase reporter gene assay (DR-CALUX; BioDetection Systems, Amsterdam, the Netherlands) as described previously (Murk et al. 1996).

### ER-mediated activity and determination of E_2_ in male blood samples.

We determined estrogenic activities of 17 prevalent PCB congeners using the ER-CALUX bioassay (BioDetection Systems) using the human breast carcinoma T47D.Luc cell line, stably transfected with pEREtataLuc construct (Legler et al. 1999; Machala et al. 2004). ER-mediated activity was also determined in the cells treated with either total serum hexane/diethyl ether extracts or with a fraction of POPs obtained by a consequent sulfuric acid/silica fractionation. We determined anti-estrogenicity as a decrease in response to E_2_ in the cells co-treated with the individual PCB or POPs fraction. Concentrations of E_2_ were determined by ELISA (ADVIA Centaur Estradiol-6 III assay; Bayer HealthCare, Tarrytown, NY, USA) in 60 samples selected according to stratified PCB levels. We determined cytotoxicity of extracts or individual PCBs by a neutral red uptake assay after a 24-hr exposure.

### Data analyses.

All calculations were performed with Microsoft Excel, SlideWritePlus 3.0 for Windows, or Statistica 6.1 for Windows (Microsoft Corporation, Redmond, WA, USA). Nonparametric statistical analyses (Kruskal-Wallis analysis of variance and the Mann-Whitney *U* test) were used for data analysis. We determined the relationships among biological and chemical data by correlation analysis and multivariate principal component analysis (PCA). We assessed the correlations among the compared parameters using nonparametric Spearman’s rank coefficient (*R*_s_). For the PCA analysis, all the data were normalized using the transformation log (*X* + 1).

## Results

**Figure 2:**
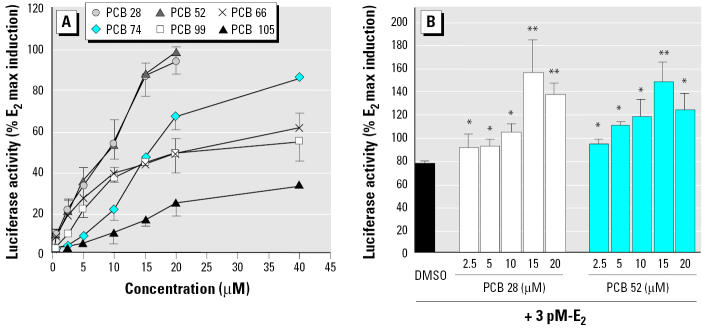
Dose-dependent estrogenic effect of the individual PCB congeners (*A*) and the combined effect of two estrogenic PCB congeners (PCBs 28 and 52) plus 3 pM E_2_ (*B*) on induction of luciferase activity in the ER-CALUX assay. Results are expressed as mean ± SD. *p < 0.05, and **p < 0.01 compared with control.

**Figure 3:**
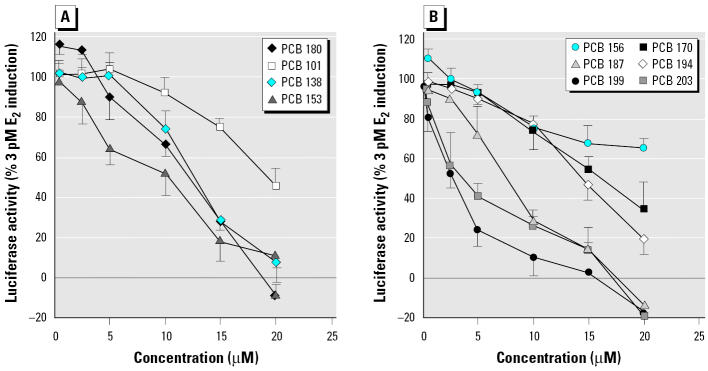
Dose-dependent effect of four indicator PCB congeners (*A*) and six higher chlorinated PCBs (*B*) on 3 pM E_2_-induced luciferase activity in T47D.Luc cells. Results are expressed as mean ± SD.

**Figure 4:**
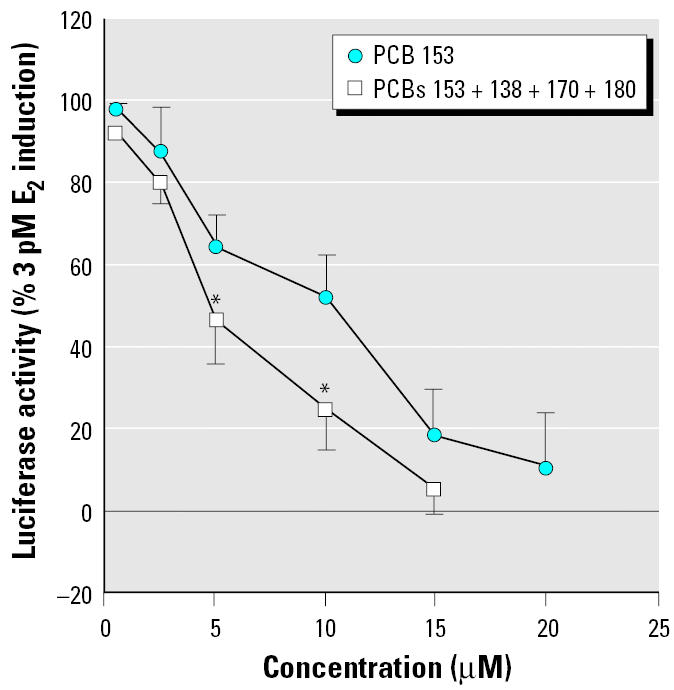
Antiestrogenic potencies of PCB 153 and an artificial mixture of the most prevalent PCBs (ratio of 6:3:5:2 of PCBs 153, 138, 170, and 180). Results are expressed as mean ± SD. ^*^p < 0.05 compared with an equimolar concentration of PCB 153.

Estrogenic and antiestrogenic potencies of a series of individual PCB congeners, found to be prevalent in the human male blood samples, were determined in the ER-CALUX assay. Lower-molecular-weight PCBs 28, 52, 66, and 74 elicited a significant ER-mediated activity at micromolar concentrations. Pentachlorobiphenyls (PCBs 99 and 105) were only partial ER agonists (Figure 2A). The ER activation by its natural ligand E_2_ was potentiated when cells were co-treated with trichlorobiphenyls (PCB 28 or 52) (Figure 2B). The most prevalent PCB congeners, 138, 153, 170, 180, and 187, as well as octachlorobiphenyls (PCBs 194, 199, and 203) did not induce the ER-dependent luciferase activity (data not shown), but they all significantly decreased the E_2_-induced luciferase activity (Figure 3). The most potent inhibitors of ER activation were PCBs 199, 203, and 153; however, the IC_50_ (concentration that inhibits 50% of maximal E_2_ response) values of all tested congeners were within a narrow concentration range, 2.9–16.0 μM. A partially reconstituted mixture of the most prevalent PCBs, reflecting a typical ratio of concentrations of individual congeners, showed a significantly higher antiestrogenic activity when compared with inhibition potency of PCB 153 (Figure 4). Potent AhR agonists (dioxin-like PCBs 126, 118, 105, and 156) did not significantly affect the ER activation in the ER-CALUX assay. The estrogenic and antiestrogenic effects of PCB congeners, including the data on their cytotoxicity and calculated median effective concentration (EC_50_) values, are summarized in Table 1.

In the second step of this study, we determined the estrogenic activities of 150 human male serum samples, collected in Michalovce and Stropkov districts, Slovakia, using the ER-CALUX bioassay. The total hexane/ diethyl ether extracts of human male serum samples, containing both endogenous steroids and POPs, showed significant estrogenic responses in the ER-CALUX assay, ranging from 12.5 to 59.2 pg E_2_ equivalents (EEQ) per milliliter (Table 2). Dioxin-like activities, measured in the POPs fractions by the DR-CALUX bioassay, ranged from 0.2 to 2.9 pg TEQs/mL (Table 2). Weak estrogenic or antiestrogenic activities were found in the fractions of POPs, but only in part of the samples. The POPs fractions from the less polluted background area elicited ER-mediated activity with a higher incidence (18 of 75 samples vs. 8 of 75 samples), whereas anti-estrogenic activity was detected more frequently in the samples from the PCB-polluted region (5 and 17 samples, respectively). However, the ER-mediated activities did not overcome 2 pg EEQs/mL, and only partial estrogenic or antiestrogenic responses (< 40%) were found in positive samples (data not shown). Conversion of concentration units showed that only submicromolar concentrations of prevalent PCBs were present in cultivation medium when serum extracts were applied to cells (data not shown); therefore, only partial antiestrogenic effects of PCBs could be expected in the sample mixtures.

The in vitro bioassay data were compared with data on concentrations of major POPs in the samples obtained in the PCBRisk project. In this large epidemiological study, > 2,000 human blood samples were evaluated for concentrations of PCBs, PCDD/PCDFs, and p,p′-DDE (Kočan et al. 2004). The analytical data for the subset of 150 male samples, which were used here for statistical analysis of in vitro bioassay data only, are summarized in Table 2. Additionally, data on induction of AhR-dependent expression of CYP1A1 and CYP1B1 mRNA in blood lymphocytes, as determined by real-time PCR (Canton et al. 2003; van Duursen et al. 2005), and E_2_ concentration determined in a substantial part of blood sample extracts were included in the statistical analysis. PCDDs did not contribute significantly to higher levels of TEQs, and concentrations of PCDFs, which might also contribute to the dioxin-like activities, were only marginally increased in highly exposed male subjects (Kočan et al. 2004). High concentrations of p,p′-DDE were found in a majority of samples (Table 2); however, only weak estrogenic and no dioxin-like activity was found for this compound (data not shown). Therefore, modulations of biological effects might be attributed mainly to differences in the concentrations of PCBs.

**Figure 5:**
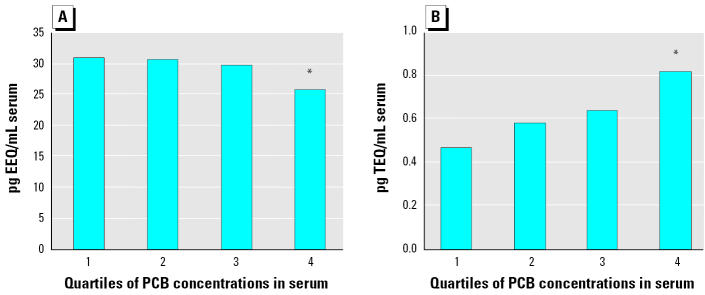
Estrogenic activities (A) and dioxin-like activities (B) of extracts of human male serum samples. Median values of quartiles were stratified according to PCB concentrations. *Significantly different from groups with lower PCB levels (p = 0.02; Mann-Whitney U test); concentrations of PCBs (μg/mL serum) are as follows: first quartile, 0.0020–0.0055; second quartile, 0.0055–0.0078; third quartile, 0.0079–0.0138; fourth quartile, 0.0139–0.1755.

Total estrogenic activity was moderately decreased, while the dioxin-like activity was increased, in samples with high PCB levels ranging from 13.9 to 175.5 ng/mL serum (i.e., 1865.7–32509.4 ng/g lipid; Figure 5). Quartiles presented in Figure 5 are based on concentrations expressed per milliliter of serum. The alternative data set (expressed on a gravimetric basis) showed a very similar pattern of effects. The levels of E_2_ decreased in the fourth quartile of PCB concentrations, but the decrease was not significant.

**Figure 6:**
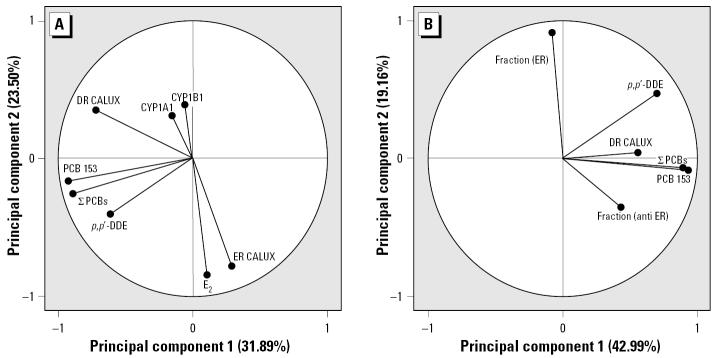
Principal component analysis of the measured parameters of the serum samples. Abbreviations: Fraction (ER), number of estrogenic samples of the fraction of POPs; fraction (anti-ER), number of anti-estrogenic samples of the fraction of POPs; ∑PCBs, serum of 17 PCB congeners.

Based on concentration data summarized in Table 2, we performed multivariate PCA to more precisely characterize statistical associations between in vitro bioassay data and levels of major organic contaminants in the blood. The PCA is one of a set of ordination techniques used in data reduction and summarization. As shown in Figure 6A, the first two components explained 55% of the variability of the original data. The axes were aligned with the directions of greatest variation in the data set. The other components were neglected because they did not contribute significantly to the meaningful interpretation of the relationships among biological and chemical parameters. The first principal component axis represented the chemical variables of the male serum extracts, such as ∑PCBs, PCB 153 concentration, p,p′-DDE content, and the biological variable AhR-mediated activity (DR-CALUX assay). The second principal component was associated with the ER-mediated activity of serum extracts (ER-CALUX assay), E_2_ concentrations, and CYP1A1 and CYP1B1 mRNA expression level. The length and direction of the lines represent the significance of the associated variables. The PCA confirmed a positive relation between overall estrogenicity (12.5–59.2 pg EEQ/ml) and E_2_ levels (1.0–43.5 pg/ml; Table 2). Further, weak negative relations between ER-mediated activity and expression of CYP1A1 and CYP1B1 mRNAs and between estrogenic and dioxin-like activities were observed (Figure 6A). The associations of the variables were confirmed by bivariate rank correlations computed on original untransformed data. The estrogenic activity of serum extracts correlated with E_2_ concentrations (R_s_ = 0.510, p < 0.001). Weak but statistically significant negative correlation between ER-mediated activity and levels of CYP1A1 mRNA (R_s_ = −0.241, p < 0.05), as well as between E_2_ concentrations and dioxin-like activity (R_s_ = −0.227, p < 0.1), were revealed. No correlation was found between E_2_ concentrations and total PCB levels (R_s_ = 0.078).

The PCA for the fractions of persistent compounds explained 60% of the total variability in the data set (Figure 6B). The first principal component was associated with a number of fractions with antiestrogenic activity (anti-ER), ∑PCBs, PCB 153 content, and AhR-mediated activity (DR-CALUX assay). The second principal component axis represented only the estrogenic activity of fractions (ER). PCA analysis showed that anti-estrogenic activity of fractions depended on the concentrations of PCB congeners and TCDD equivalents obtained in the DR-CALUX assay (R_s_ = 0.246–0.275, p < 0.01).

## Discussion

PCBs have been reported to be both estrogenic and antiestrogenic, based on various in vitro and in vivo models. Lower-molecular-weight PCBs are reportedly estrogenic, with the exception of dioxin-like 3,3′,4,4′-tetrachlorobiphenyl (PCB 77), which elicited anti-estrogenicity in vivo and also in some in vitro models (Ramamoorthy et al. 1999). 2,2′,6,6′-Tetrachlorobiphenyl (PCB 54), a fully ortho-substituted compound not occurring in the environment at significant levels (Hansen 1998), was estrogenic both in the MCF-7 cell focus assay and in the rat uterotrophic assay (Arcaro et al. 1999). 3,3′,5,5′-Tetrachlorobiphenyl (PCB 80), another model congener, was a weak ER agonist both in vivo and in vitro, while, surprisingly, PCB 52 was inactive in the same models (Nesaretnam and Darbre 1997). PCB 66 and PCB 95 (2,2′,3,5′,6-pentachlorobiphenyl) have been reported to be estrogenic in BG1LucE2 cells at 10 μM concentrations, whereas coplanar PCB 77 elicited no ER-mediated activity in this cellular model (Rogers and Denison 2000). PCB 52 and PCB 77 caused a modest transient uterotrophic effect in weaning rats (Rose et al. 2002); however, PCB 77 attenuated the increase in uterine weight and cell proliferation in another study (Jansen et al. 1993). The uterotrophic effects after exposure to less persistent PCB congeners showed nonlinear dose responses, and they decreased rapidly (Rose et al. 2002). However, all the above data have been obtained from various models and assays, and estrogenic/ antiestrogenic effects of both lower chlorinated and higher chlorinated PCBs present in the environment have not yet been examined systematically in one assay.

In our study, PCBs 28, 52, 66, 74, 99 and 105, all found at significant levels in male serum samples, induced the ER-mediated activity at micromolar concentrations (Table 1), suggesting that ER activation could be one of the potential modes of action of low-molecular-weight PCBs. However, the decrease of total estrogenic activity and E_2_ levels observed in human serum samples of males exposed to high PCB levels (Figure 5A) indicated that PCB mixtures elicited an overall antiestrogenic effect. Therefore, the ER-mediated activity of lower-chlorinated PCBs appears to have only a limited toxicologic significance, perhaps with the exception of acute transient exposure to PCBs (Rose et al. 2002).

Unlike low-molecular-weight PCBs, the dioxin-like and prevalent high-molecular-weight PCB congeners are considered to be antiestrogenic. TCDD, a model toxicant for dioxin-like PCBs, exhibits potent anti-estrogenic activity (Buchanan et al. 2000, 2002; Cooke et al. 2001; Safe and Wörmke 2003). TCDD has little effect on total ER levels (Gierthy et al. 1996), and no direct binding to ER has been reported (see Safe and Wörmke 2003). Recently, inhibition of ER-mediated cell proliferation by coplanar PCBs has been reported in breast cancer cell lines (Oenga et al. 2004). TCDD or coplanar PCBs did not inhibit E_2_-induced activity of a reporter construct containing the promoter insert from creatine kinase B in T47D cells, while dioxin-like compounds, including PCB 77 and PCB 126, prevented activation of other reporter constructs in both MCF-7 and T47D cells, although only at levels as high as 10 μM (Ramamoorthy et al. 1999). This suggests that a type of reporter construct can affect detection of antiestrogenic activity. One possible mechanism of antiestrogenic activity of AhR ligands is the direct inhibition of E_2_-responsive genes through binding to inhibitory dioxin responsive elements (iDRE) in their promoter regions. Functional iDREs have been identified in promoter regions of pS2, c-fos, Hsp27 and cathepsin D genes (reviewed by Safe and Wörmke 2003). In the present study, anti-estrogenicity was not elicited by coplanar PCB 126 (Table 1) in the T47D.Luc cells used in the ER-CALUX assay. The lack of anti-estrogenic activity of coplanar PCBs observed in the T47D.Luc cell line might be explained by the missing iDREs in the reporter construct, which contains three tandem repeats of the consensus estrogen-responsive element (ERE) oligonucleotide (Legler et al. 1999).

On the other hand, this cellular model allowed us to investigate a direct activation of ER and/or perturbation of E_2_-induced ER activation. While the low-molecular-weight PCBs elicited ER activation and ER-dependent gene expression, prevalent and more persistent high-molecular-weight PCB congeners were antiestrogenic (Table 1, Figures 3 and 4). Pulses of exposure to more labile mixtures of lower chlorinated PCBs may contribute to transient endocrine disruption, including an increase in estrogenic activity (Hansen 1998; Rose et al. 2002). PCB 153 was estrogenic in the acute 2-day immature rat uterine weight assay, albeit at very high concentrations (Li et al. 1994). Antiestrogenic effects of three prevalent congeners (PCBs 138, 153, and 180) have been found both in a reporter gene and cell proliferation MCF-7 assays (Bonenfeld-Jorgensen et al. 2001). This is in accordance with our data on antiestrogenicity of high-molecular-weight PCBs in the ER-CALUX assay (Table 1). Inhibition of ER activation by hexa-, hepta- and octachlorinated biphenyls and suppression of estrogenic signaling found in serum of males chronically exposed to PCBs (Figures 3–5) suggest that PCB 153 and other prevalent congeners could contribute to overall antiestrogenic response. Contribution of hydroxy- and methylsulfonyl-PCB metabolites, p,p′-DDE and methylsulfonyl-p,p′-DDE metabolites to antiestrogenic activities of POPs might also be of importance. Because both low- and high-molecular-weight PCBs and POPs metabolites elicited their effects on estrogenic activity at similar micromolar concentrations (Bonenfeld-Jorgensen et al. 2001; Letcher et al. 2002; Machala et al. 2004; this study), it might be expected that the anti-estrogenic effects of prevalent higher chlorinated PCBs would prevail in human male blood. Nevertheless, antiestrogenic effects of PCBs detected in human male serum appeared to be less important, when compared with the levels of E_2_, the major contributor of the overall estrogenic activity.

In vitro bioassays are a suitable tool for exposure assessment of dioxin-like and (anti)estrogenic compounds (van den Berg et al. 1998; Zacharewski 1997). However, currently only limited data are available on dioxin-like activities found in human female serum and follicular fluid; the TEQs determined by the DR-CALUX assay have been reported to correlate well with the sum of four major PCB congeners 153, 138, 180, and 118 (Pauwels et al. 2000). The data on the ER-mediated activity in human blood samples are still limited. Rasmussen et al. (2003) reported estrogenic activity in female serum by E-Screen assay, however, they did not observe any correlation between estrogenicity and concentrations of individual endocrine disruptors. In the present study, a decrease of total estrogenic activity and increased dioxin-like activity were found in serum samples from human males chronically exposed to PCBs. However, as shown in Figure 5, correlations with PCB concentrations were significant only in subjects with high exposure levels.

Our data suggest that exposure to high levels of PCBs might affect E_2_ blood levels, although this was not significant. Currently, there is little information available about a possible modulation of steroid hormone levels after exposure to PCBs. In a recently published study, a weak but significant negative correlation was found between serum levels of the prevalent PCB 153 congener and testosterone in young men, and E_2_ concentrations (within a concentration range of 43–144 pM) were also slightly decreased in the more exposed subjects (Richthoff et al. 2003). The concentrations of PCB 153 (23–250 ng/g lipid) found in these subjects were significantly lower that those observed both in the present study and in previous studies in eastern Slovakia (Kočan et al. 2001, 2004). The concentrations of PCB 153 ranged from 115 to 8,631 ng/g lipid in 150 Slovak male serum samples included in the present study. Another experimental study in rats exposed to PCB mixtures also reported lower testosterone and E_2_ serum levels and suppression of brain aromatase activity (Hany et al. 1999). Decreased E_2_ concentrations could be associated with AhR activation by dioxin-like PCBs, leading to enhanced CYP1A/CYP1B1-catalyzed metabolism of E_2_ (Gierthy et al. 1996; Spink et al. 1990). Induction of CYP1A1 and CYP1B1 mRNAs in lymphocytes is considered to reflect increased exposure to dioxin-like compounds (Canton et al. 2003; Hanaoka et al. 2002). Within the epidemiologic study, Canton et al. (2004) found increased levels of CYP1A1 and CYP1B1 mRNA only in lymphocytes of males exposed to very high levels of PCBs (fourth quartile). This finding suggests that a physiologically significant AhR-dependent induction of E_2_-metabolizing CYP enzymes might occur in liver and other target tissues.

Besides CYP1A1, 1A2, and 1B1 iso-enzymes, CYP3A4 has been suggested to play a major role in hydroxylation of E_2_ (Badawi et al. 2000; Hayes et al. 1996; Pang et al. 1999; Spink et al. 1990; Takemoto et al. 2004; Yamazaki et al. 1998). Induction of CYP3A4 is a consequence of exposure to prevalent nondioxin-like PCBs (Gillette et al. 2002; Parkinson et al. 1981). Therefore, both coplanar and noncoplanar PCBs could increase E_2_ metabolism and reduce blood E_2_ concentrations.

**Figure 7:**
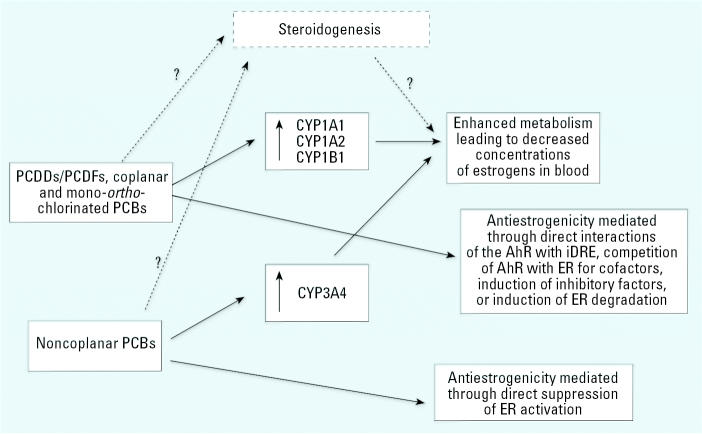
Potential mechanisms of antiestrogenic effects of coplanar and noncoplanar PCBs.

Both dioxin-like and nondioxin-like PCBs might affect estrogen signaling by multiple mechanisms, as summarized in Figure 7. Obviously, this list of modes of action is not complete; PCBs might also potentially disrupt the pathways associated with the perturbation of hypothalamus–pituitary–gonadal axis hormone signaling and steroidogenesis, as another potential mechanism of E_2_ modulations by PCBs.

As outlined in the recent review by Safe (2004), it is currently not possible to directly attribute increased incidence of breast cancer or disorders of the male reproductive tract, for example, to endocrine disruption associated with organochlorine exposure. A number of adverse impacts of high PCB contamination have been identified, including perturbations of thyroid function, immunity, or neuro-developmental processes (Robertson and Hansen 2001). However, it was not possible to associate any of the adverse effects observed within the frames of the PCBRisk project with antiestrogenicity of PCBs.

In summary, significant associations between exposure to PCBs and overall (anti)estrogenic and dioxin-like activities in the present study were found only at high exposure levels. Although the prevalent noncoplanar PCBs elicited antiestrogenicity in the ER-CALUX assay, when tested as individual compounds or as a partially reconstituted mixture, a significant estrogenic activity was determined in whole-serum extracts. Moreover, only weak or negligible anti/estrogenic activities were found in serum extract fractions containing exclusively POPs including PCBs. Due to the presence of E_2_ in human male blood and its dominant role in total estrogenic activity of serum samples, reduction of E_2_ levels might be a more significant antiestrogenic effect of high PCB exposure. This mode of action, associated with induction of CYP1A1, CYP1A2, CYP1B1 and/or CYP3A4 enzymes or perturbation of steroidogenesis and endocrine signaling, preceding the biosynthesis of estrogens, deserves further attention.

## Figures and Tables

**Table 1:** PCB congeners under study, including molecular weights and estrogenic/antiestrogenic and cytotoxic effects determined in ER-CALUX assay using human breast carcinoma T47D.Luc cells.

		ER-activated activity		Antiestrogenicity		
PCB (IUPAC)	Structure	IEC^a^ (μM)	IEF^b^	IC_50_^c^ (μM)	IhEF^d^	Cytotoxicity LOEC^e^ (μM)
28	2,4,4′-Trichlorobiphenyl	8.23	1.65 × 10−7	NI	NI	> 20
52	2,2′,5,5′-Tetrachlorobiphenyl	9.52	1.42 × 10−7	NI	NI	> 20
66	2,3′,4,4′-Tetrachlorobiphenyl	24.31	8.56 × 10−8	NI	NI	> 40
74	2,4,4′,5-Tetrachlorobiphenyl	17.00	1.24 × 10−7	NI	NI	> 40
99	2,2′,4,4′,5-Pentachlorobiphenyl	WI	WI	NI	NI	> 40
105	2,3,3′,4,4′-Pentachlorobiphenyl	WI	WI	NI	NI	> 40
118	2,3′,4,4′,5-Pentachlorobiphenyl	NI	NI	NI	NI	> 20
126	3,3′,4,4′,5-Pentachlorobiphenyl	NI	NI	NI	NI	> 40
138	2,2′,3,4,4′,5′-Hexachlorobiphenyl	NI	NI	10.12	4.94 × 10−6	20
153	2,2′,4,4′,5,5′-Hexachlorobiphenyl	NI	NI	5.89	8.50 × 10−6	20
156	2,3,3′,4,4′,5-Hexachlorobiphenyl	NI	NI	WI	WI	40
170	2,2′,3,3′,4,4′,5-Heptachlorobiphenyl	NI	NI	16.03	3.12 × 10−6	25
180	2,2′,3,4,4′,5,5′-Heptachlorobiphenyl	NI	NI	9.32	5.36 × 10−6	20
187	2,2′,3,4′,5,5′,6-Heptachlorobiphenyl	NI	NI	7.48	6.68 × 10−6	20
194	2,2′,3,3′,4,4′,5,5′-Octachlorobiphenyl	NI	NI	14.14	3.54 × 10−6	25
199	2,2′,3,3′,4′,5,6,6′-Octachlorobiphenyl	NI	NI	2.85	1.75 × 10−5	20
203	2,2′,3,4,4′,5,5′,6-Octachlorobiphenyl	NI	NI	3.20	1.56 × 10−5	20

Abbreviations: IEC, induction equivalency concentration; IEF, induction equivalency factor; IhEF, inhibitory equivalency factor; NI, no significant induction/inhibition; WI, weak induction/inhibition (< 50% of estradiol maximum induction; < 50% decrease in induction of 3 pM E_2_).

a Concentration of PCB congener inducing the same level of luciferase activity as the EC_50_ of the reference inducer E_2_ (2.08 pM).

b Calculated as the ratio between the EC_50_ of E_2_ and the concentration of the selected PCB congener inducing the same level of luciferase activity.

c Concentration of PCB congener causing 50% decrease in luciferase activity induced by 3 pM E_2_.

d Calculated as the ratio between the IC_50_ of the synthetic antiestrogen ICI 182,780 (IC_50_ = 50 pM) and the concentration of the selected PCB congener causing the same level of decrease in luciferase activity induced by 3 pM E_2_.

e Lowest (experimental) concentration of PCB congener causing a significant decrease of cell viability (24-hr exposure Neutral Red uptake assay).

**Table 2:** Summary of data from human male serum samples used in multivariate statistical analysis.

		Concentration/mL serum			Concentration/g lipid		
	No.	Range	Median	Mean	Range	Median	Mean
Estrogenic activity (pg EEQs)	150	12.5–59.2	28.2	29.2	1.3–11.6	4.1	4.3
E_2_ (pg)	60	< 1.0–43.5	15.5	15.8	0.1–5.4	2.0	2.1
Dioxin-like activity (pg TEQs)	144	0.2–2.9	0.6	0.7	11.9–434.0	83.6	92.0
∑ PCBs/PCDD/PCDFs (pg TEQs)	100	0.05–0.5	0.1	0.2	7.5–57.9	18.2	20.8
∑ PCBs (μg)	150	0.0020–0.1755	0.0078	0.0147	0.3458–32.51	1.124	2.040
p,p′-DDE (μg)	150	0.0017–0.1165	0.0119	0.0171	0.2689–11.16	1.800	2.219

Sum of PCDD/PCDFs and dioxin-like PCBs was calculated as TEQs according to World Health Organization TEF values (van den Berg et al. 1998).
